# Acute Traumatic Musculotendinous Avulsion of the Flexor Pollicis Longus Tendon Treated with Primary Flexor Digitorum Superficialis Transfer: A Novel Technique of Management

**DOI:** 10.1155/2016/2106203

**Published:** 2016-02-25

**Authors:** P. Kiran Sasi, Swagath Mahapatra, Samuel C. Raj Pallapati, Binu P. Thomas

**Affiliations:** Dr. Paul Brand Center for Hand Surgery, Christian Medical College and Hospital, Vellore 632004, India

## Abstract

Traumatic musculotendinous junction avulsions are rare injuries except in avulsion amputations. They pose a significant challenge to the treating surgeon. We present a 24-year-old male who sustained an open musculotendinous avulsion of the flexor pollicis longus tendon. He was treated with primary tendon transfer using the flexor digitorum superficialis of ring finger, in flexor zone 3. The functional result at 10 months following surgery was excellent.

## 1. Introduction

The weakest parts of the muscle-tendon unit are musculotendinous junction and bony insertion. Avulsions from bony insertion are common and have been studied extensively. But avulsions from musculotendinous junction are rare injuries except in avulsion amputations. There are only 7 reports of closed flexor tendon ruptures at the musculotendinous junction [1–3]. Three of them involved the flexor digitorum superficialis tendon and the remaining four involved the flexor pollicis longus tendon [1–3]. These injuries are unique because of the difficulty in management and also the expected functional outcome.

We present a case of traumatic musculotendinous avulsion of the flexor pollicis longus (FPL) tendon resulting from extreme longitudinal force applied to the tendon after an open hand injury. The chosen technique for primary tendon transfer led to a very good functional outcome.

## 2. Case History

**Figure 1:**
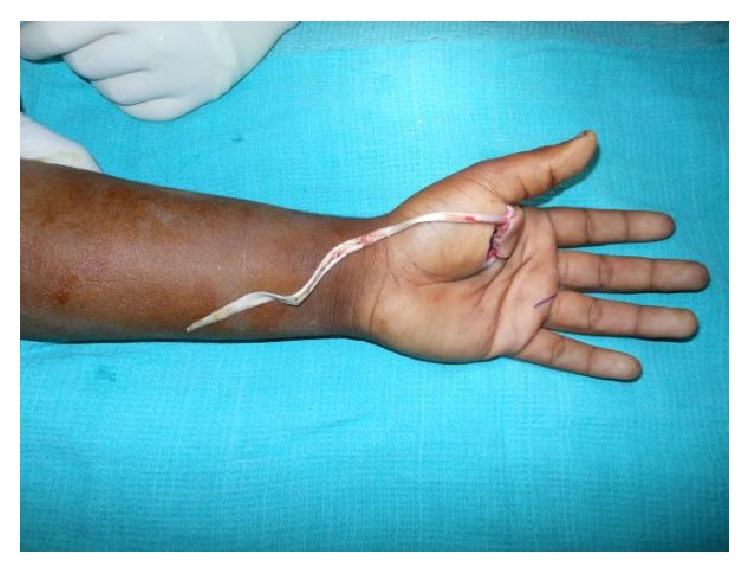
Traumatic avulsion of the flexor pollicis tendon from the musculotendinous junction.

**Figure 2:**
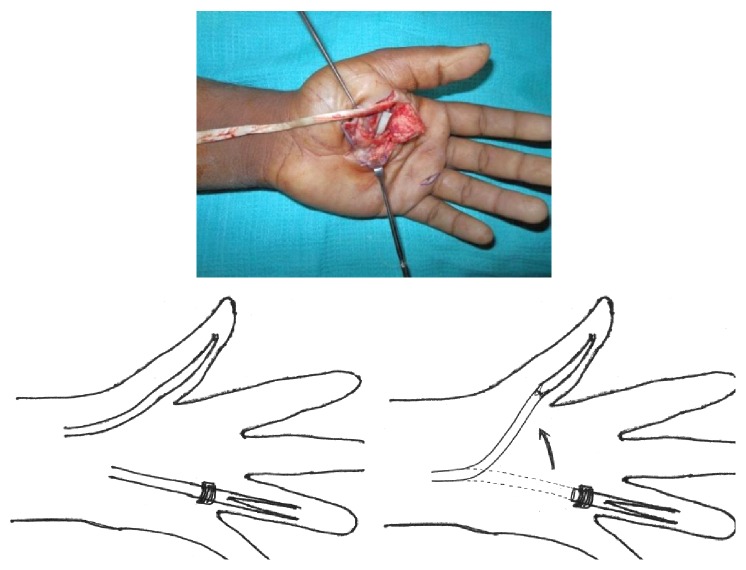
Flexor digitorum superficialis tendon from the ring finger harvested and tunnelled deep to the neurovascular bundle.

**Figure 3:**
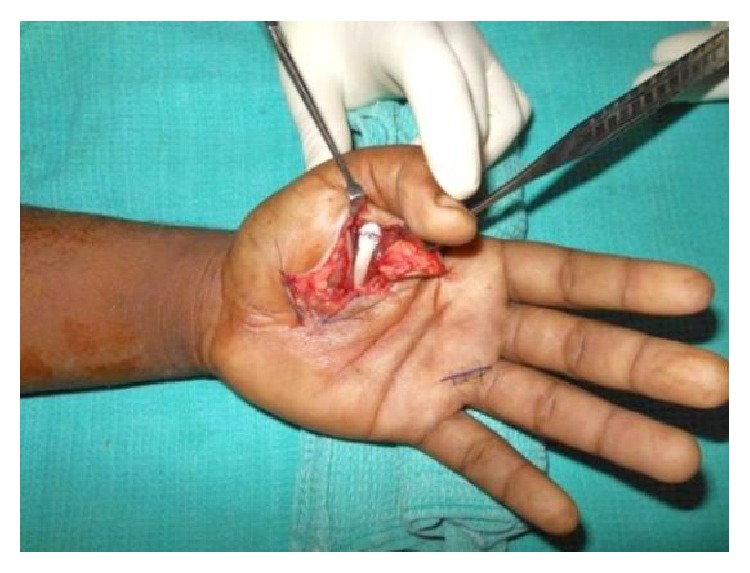
Flexor digitorum superficialis tendon repaired to the flexor pollicis tendon.

**Figure 4:**
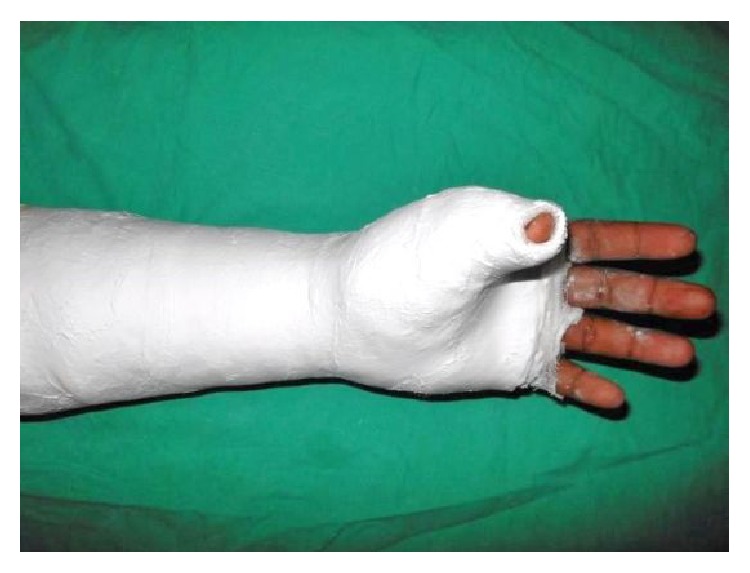
Post-op thumb spica cast applied.

**Figure 5:**
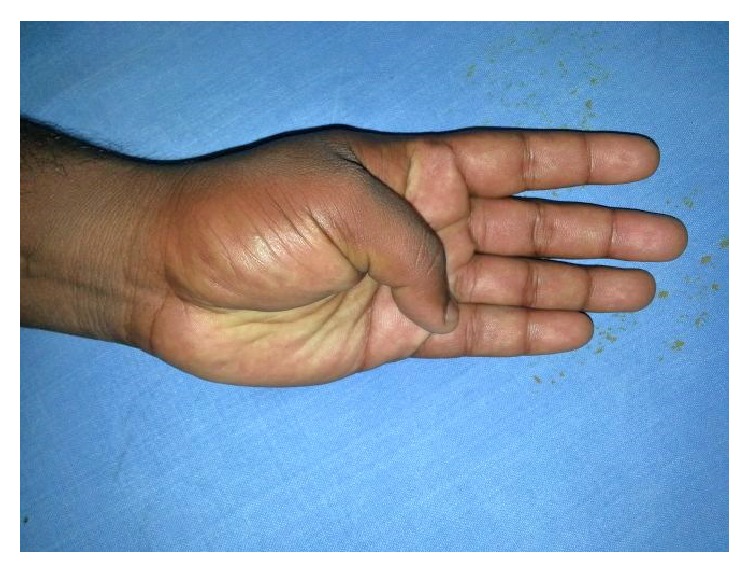
Follow-up showing thumb reaching the little finger basal crease.

**Figure 6:**
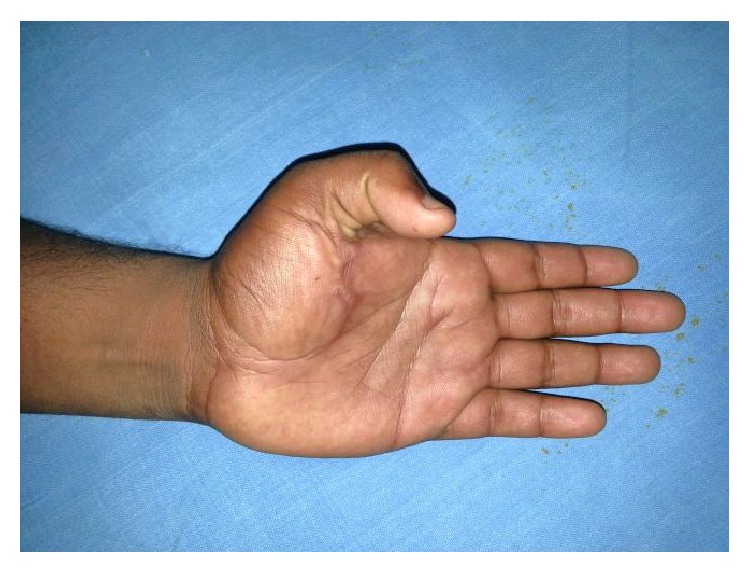
Follow-up showing full interphalangeal joint flexion.

**Figure 7:**
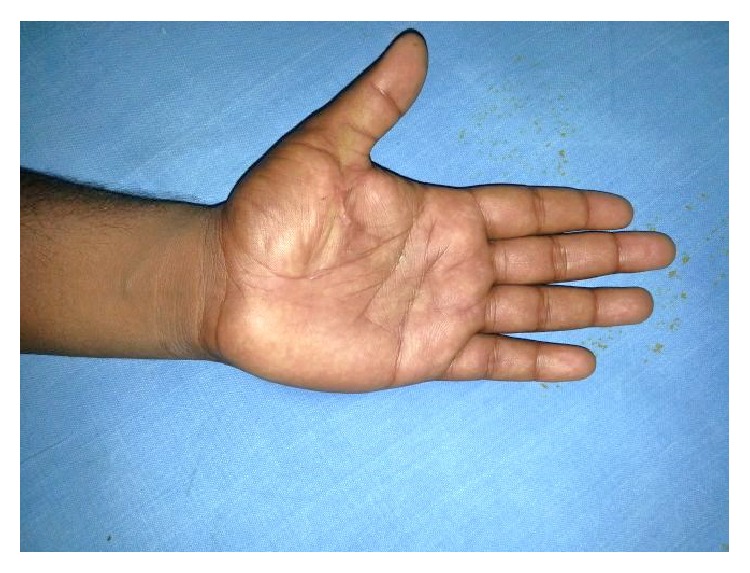
Follow-up, no extension lag.

**Figure 8:**
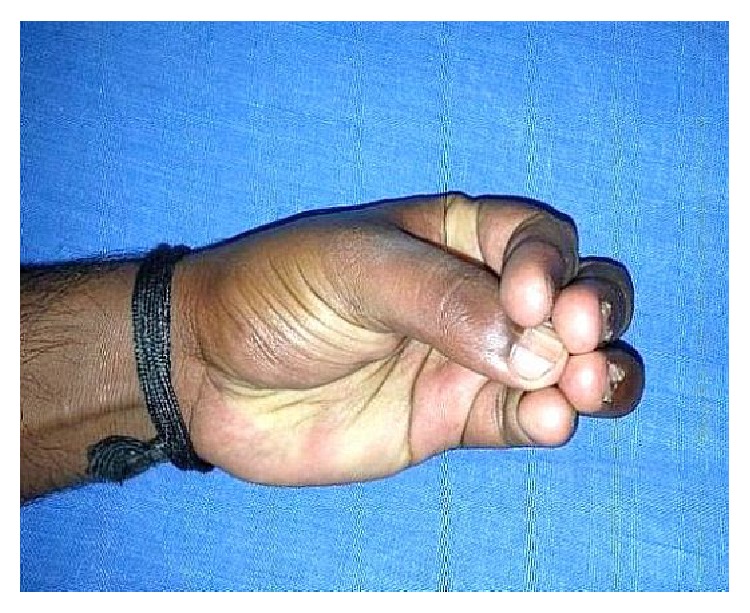
Follow-up, normal pulp pinch.

A 24-year-old male sustained direct trauma to the left hand when his hand got caught in a rusted sharp metal object. While in a religious procession, he tried to reach up and touch an idol when he fell and the entire weight of his body was suspended at that point. This resulted in complete avulsion of the FPL tendon from its muscle belly (Figure 1). He did not sustain any other injuries. He presented to our hospital 12 hours after the initial injury and was immediately taken to the minor operating room in Accident and Emergency. Under wrist block, wound debridement and copious irrigation were carried out. IV antibiotics and tetanus prophylaxis were given. It was decided to perform a primary tendon transfer considering the significant delay in presentation and the fact that flexor pollicis longus is a unipennate muscle might not respond well to proximal repair. The flexor digitorum superficialis of ring finger (FDS-R) was chosen as the donor. Supraclavicular brachial block was given and tourniquet was inflated after administration of intravenous antibiotics. Thorough wound debridement and copious irrigation were done. Proximal forearm was not explored. An incision was made at the volar aspect of the palm to access the FDS-R. The tendon was divided proximal to the A1 pulley and tunnelled under the superficial palmar arch and branches of the median nerve to the thenar wound (Figure 2). Distal stump of the FPL tendon end was debrided. Tenodesis test was done where interphalangeal joint flexion was assessed in different wrist positions and appropriate length of the tendon was identified. End-to-end repair of FDS-R to distal FPL tendon was done using 4-strand core sutures using 4-0 Ethibond and running continuous epitendinous sutures using 6-0 Ethilon (Figure 3). Wound drain was placed and skin was closed with Ethilon 4-0. Short arm thumb spica cast was applied (Figure 4). Injection of Cefazolin was administered for 2 days. Supervised passive mobilization was started at 4 weeks and active mobilization was started at 6 weeks. The wounds healed uneventfully and no complications were observed. Thumb movement at both the metacarpophalangeal and interphalangeal joints returned very close to normal within 4 months (full flexion, 5° extension deficit of the interphalangeal joint of the involved side, and full opposition). Key, pulp, and tripod grip strengths were tested with a digital dynamometer and reached 80%, 95%, and 90% of the strength of the uninjured side, respectively, by 5 months. On final follow-up evaluation 7 months after surgery, the patient was able to reach the basal crease of the little finger with the tip of the thumb (Figure 5); there was full flexion (Figure 6) and extension (Figure 7) at the interphalangeal joint. He was able to do good pulp pinch (Figure 8) and power grip. The outcome as evaluated according to the criteria of Fitoussi et al. [4] (Table 1) was excellent with a score of 9 of 9.

The ring finger did not develop superficialis-minus deformity or swan neck deformity.

## 3. Discussion

Disruption of a muscle-tendon unit can occur at any of these five areas along its length: (1) the bony insertion of the tendon, (2) the midsubstance of the tendon, (3) the musculotendinous junction, (4) the muscle belly, or (5) the origin of the muscle [1]. Among these, the weakest link is the bony insertion followed by the musculotendinous junction [1, 2, 5] with the former accounting for 62.8% of cases and the latter only 5.1%, in Boyes' series of 78 tendon ruptures [2]. Avulsion of FPL is common only during avulsion amputations of the thumb and, commonly, occurs together with avulsion of extensor pollicis longus [3, 6]. Rupture of FPL tendon is the first recorded flexor tendon rupture and was reported as early as 1891 [2]. Despite that, there is sparse description of this injury. In the event of an injury, restoring the function of FPL demands utmost care because thumb provides more than 40% of the entire hand function [7]. Side-to-side suturing of FPL to flexor digitorum profundus tendon of the index finger has been described but this results in their concomitant movement [8], which is undesirable. A technique described for acute open musculotendinous avulsion of direct repair by encapsulation of the tendon into the muscle belly has been described. But this was performed in a young patient who presented early and was operated upon within the golden hour of trauma [9]. This method is not widely considered to be a strong repair and is favoured only in situations of avulsion amputation [1]. Primary tendon transfer with FDS-R is an excellent option in especially in middle-aged and elderly patients. This method is particularly useful in situations where there is considerable delay in presentation which will lead to tendon dessication.

Superficialis-minus and subsequent swan neck deformity are seen in about 15% of cases of superficialis harvest for tendon transfer [10]. When the tendon is harvested proximal to A1 pulley, the digit will not develop these deformities.

The paucity of such description in known English literature makes this a unique and novel method of managing similar injuries.

## Figures and Tables

**Table 1:** Fitoussi et al. criteria for flexor pollicis longus recovery.

	Degrees	Points
Flexion of IP joint noninjured side, flexion of IP joint of involved side	0 to 20	6
	21 to 40	4
	41 to 50	2
	>50	0

Extension deficit (comparison with contralateral side)	0 to 10	3
	11 to 20	2
	21 to 30	1
	>30	0

Evaluation		
Excellent	8-9	
Good	6-7	
Fair	4-5	
Poor	0–3	
